# Ultrasound biomicroscopy and anterior segment OCT in ocular surface squamous neoplasia: complementary roles, case series, and targeted literature review

**DOI:** 10.3389/fopht.2026.1794093

**Published:** 2026-03-12

**Authors:** Miri Meira Fogel Levin, Iris Moroz, Yael Birger, Avner Hostovsky, Shalev Fried, Ido Didi Fabian, Vicktoria Vishnevskia Dai, Daphna Landau Prat, Guy J. Ben Simon

**Affiliations:** 1 Goldschleger Eye Institute, Sheba Medical Center, Tel-Hashomer, Israel; 2 Gray Faculty of Medicine, Tel Aviv University, Tel Aviv, Israel

**Keywords:** anterior segment optical coherence tomography, intraocular extension, ocular surface squamous neoplasia, scleral invasion, ultrasound biomicroscopy

## Abstract

**Introduction:**

Ocular surface squamous neoplasia (OSSN) encompasses a spectrum of epithelial malignancies of the conjunctiva and cornea that, untreated, may invade intraocularly or into the orbit. Clinical examination alone may fail to define true depth of invasion, risking under- or overtreatment. Advanced anterior segment imaging provides objective structural information that complements slit-lamp assessment.

**Methods:**

This review summarizes the complementary roles of anterior segment optical coherence tomography (AS-OCT) and ultrasound biomicroscopy (UBM) in assessing OSSN depth through a targeted literature review and illustrates their practical applications through representative cases from a single tertiary center.

**Results:**

AS-OCT offers non-contact, high-resolution imaging of the epithelium and stromal interface, facilitating detection of invasive change. UBM, with deeper penetration, visualizes the sclera, ciliary body, angle, and iris, detecting deep or clinically occult extension. The cases demonstrate how each imaging modality contributes to refining diagnosis, guiding treatment selection, and supporting long-term surveillance.

**Discussion:**

Applying AS-OCT for superficial disease and UBM for suspected deep extension enhances diagnostic confidence and supports personalized, globe-preserving management. Integrating both modalities into a stepwise imaging workflow can improve staging accuracy and align treatment intensity with true disease extent.

## Introduction

Ocular surface squamous neoplasia (OSSN), the most common non-pigmented malignancy of the ocular surface, encompasses a spectrum of malignant and premalignant epithelial lesions affecting the conjunctiva and cornea (1, 2). Although typically non-pigmented, OSSN may occasionally present as a pigmented lesion and can mimic melanocytic pathology (2). The spectrum ranges from conjunctival intraepithelial neoplasia (CIN; grades 1-3), where dysplastic cells remain confined to the epithelium, to invasive squamous cell carcinoma (SCC) with stromal penetration (1). While most cases present as localized disease amenable to topical chemotherapy or simple surgical excision, a critical subset develops deep extension with scleral invasion, intraocular involvement, or orbital extension (3, 4). These advanced cases are associated with reduced five-year disease-free survival and may require aggressive interventions including orbital exenteration and adjuvant radiotherapy (4–6); notably, some ocular oncology centers recommend adjuvant radiotherapy for all invasive conjunctival SCC. Intraocular extension often presents subtly, mimicking benign inflammatory conditions and contributing to delayed recognition; although uncommon, it is clinically significant, reported in approximately 2–12% of invasive cases, and prior intraocular surgery has been described as a potential route of spread through surgical wounds (7, 8).

OSSN spreads primarily by direct extension, and therefore anterior segment imaging studies are often required to assess the extent of invasion. Clinical examination demonstrates significant limitations in detecting deep invasion, with diagnostic accuracy of 40-86% compared to histopathology and inability to reliably determine tumor depth or assess posterior margins (9, 10). Given these limitations, advanced anterior segment imaging with ultrasound biomicroscopy (UBM) and anterior segment optical coherence tomography (AS-OCT) has become increasingly valuable for OSSN assessment, providing complementary capabilities that overcome the inherent constraints of clinical examination alone.

This review examines the complementary roles of UBM and AS-OCT in detecting and characterizing deep OSSN extension, discussing mechanisms, clinical applications, and the comparative advantages of each modality. In addition, we present representative cases in which AS-OCT and UBM were applied to delineate tumor extent.

## Methods

This manuscript combines a targeted literature review with representative cases illustrating the clinical application of anterior segment imaging in OSSN.

### Literature review

A targeted review of the English-language literature was performed using PubMed and Google Scholar. Search terms included “ocular surface squamous neoplasia”, “OSSN”, “anterior segment OCT”, “AS-OCT”, “ultrasound biomicroscopy”, and “UBM”, Relevant articles addressing imaging characteristics, diagnostic accuracy, and clinical applications were reviewed and synthesized.

### Case selection

Cases were identified retrospectively at Sheba Medical Center, Goldschleger Eye Institute between 2015 and 2024. This is a convenience sample intended to provide practical illustrations rather than a consecutive cohort. All cases were histopathologically confirmed. Inclusion criteria were diagnosis of OSSN with interpretable anterior segment imaging (AS-OCT and/or UBM). Cases with insufficient imaging quality were excluded. Imaging modality selection was clinician-driven based on clinical presentation without a predefined institutional protocol.

### Imaging acquisition

AS-OCT was performed on a Heidelberg Spectralis platform (Heidelberg Engineering, Germany) with an anterior segment lens module. UBM was performed on the Quantel Medical ABSOLU platform (Quantel Medical, France) using a water-filled balloon technique.

### Institutional management approach

At our institute, ocular oncology and oculoplastics collaborate in the multidisciplinary management of ocular surface tumors. All lesions undergo complete surgical excision with cryotherapy to the margins. Larger lesions or those involving the corneal surface are reconstructed with amniotic membrane grafting. Adjuvant topical chemotherapy is administered when surgical margins are involved or when incomplete excision is suspected. Brachytherapy is considered for invasive disease, and systemic immunotherapy may be added when there is concern that brachytherapy alone may not provide adequate coverage. Imaging-based staging informs surgical planning and treatment intensity.

## Review of imaging modalities

### Anterior segment optical coherence tomography in OSSN

AS-OCT employs near-infrared light to generate high-resolution cross-sectional images of the ocular surface in a non-contact manner. The technology achieves superior axial resolution of 2–7 micrometers with tissue penetration of 2–3 mm (11). AS-OCT’s non-invasive nature, rapid image acquisition, and exceptional resolution for superficial structures make it the preferred first-line imaging modality for OSSN diagnosis and treatment monitoring.

### Diagnostic features and imaging characteristics

AS-OCT demonstrates characteristic findings in OSSN that enable differentiation from benign ocular surface lesions. The classic diagnostic triad consists of hyperreflective epithelium, abrupt transition between normal and abnormal tissue, and marked epithelial thickening (12–16). These features show excellent correlation with histopathological characteristics and demonstrate high specificity for distinguishing OSSN from benign conditions such as pterygium or inflammatory lesions.

The hyperreflectivity pattern observed in OSSN correlates with increased keratinization and cellular density characteristic of dysplastic and neoplastic epithelium (12, 13, 16). This increased reflectivity contrasts sharply with the relatively hyporeflective appearance of normal conjunctival epithelium and the fibrovascular tissue of pterygium.

The abrupt transition zone between normal and abnormal epithelium represents another key diagnostic feature, manifesting as a sharp demarcation between areas of normal thickness with low reflectivity and thickened, hyperreflective tumor epithelium (12, 13). This contrasts with the gradual transition typically observed in inflammatory or degenerative conditions.

Epithelial thickness measurements represent the most robust quantitative parameter for OSSN diagnosis. Multiple studies establish significantly greater mean epithelial thickness in OSSN (295–346 micrometers) compared to pterygium (80–101 micrometers) or normal conjunctiva (12, 15, 16). Proposed diagnostic cutoffs range from 97–142 micrometers, achieving sensitivity of 87-100% and specificity of 94.7-100% for OSSN detection (12, 16, 17).

AS-OCT demonstrates a visible plane of cleavage between the lesion and underlying substantia propria in intraepithelial disease, with loss of this clear tissue plane suggesting stromal invasion (18). This finding helps distinguish between CIN and invasive SCC, with important implications for management. When OSSN involves the cornea, AS-OCT enables detailed visualization of lamellar architecture and can quantify invasion depth, providing critical information for surgical planning and prognosis determination (14, 18, 19).

### Other clinical applications and limitations

Serial imaging enables monitoring response to topical chemotherapy through objective quantification of epithelial thickness reduction, normalization of hyperreflectivity, and restoration of the epithelial-stromal demarcation (13, 20, 21). The non-invasive nature permits frequent assessment for both treatment monitoring and long-term surveillance for recurrence (11, 14).

Significant limitations exist in deep tissue assessment due to the 2–3 mm maximum penetration depth. While normal anterior scleral thickness ranges from 682 micrometers to 818 micrometers (at 1 mm to 6 mm from the limbus) (22), AS-OCT cannot reliably detect deep scleral invasion, particularly in pigmented lesions where light scattering further limits penetration (14, 23). Similarly, although AS-OCT provides superior visualization of the anterior chamber angle architecture and can detect hyperreflective deposits suggesting anterior chamber involvement (24, 25), it cannot penetrate beyond the pigmented iris posterior surface. Therefore, suspected deep scleral invasion or intraocular extension requires correlation with UBM for complete assessment of the sclera and ciliary body (14, 23, 25).

### Ultrasound biomicroscopy in OSSN

UBM is a high-frequency ultrasound modality that provides ~4–5 mm of tissue penetration for cross-sectional imaging of the anterior sclera, ciliary body, and angle, including structures posterior to the iris (26–28). It plays an essential role in detecting deep invasion by OSSN that may not be clinically evident; in a series of seven cases with intraocular extension, invasion was identified in four despite an unremarkable clinical examination (8).

### Tumor characterization and scleral assessment

Typical sonographic features include a hyperechoic tumor surface with hypoechoic stroma, distinguishable from the generally more hyperechoic appearance of adjacent orbital tissues (8, 29). The bright superficial layer likely represents keratinized epithelium, whereas the darker internal signal reflects the cellular tumor stroma.

Tumor height measured by UBM correlates strongly with invasion risk. OSSN with intraocular extension demonstrates a mean tumor height of 5.06 mm compared to 2.93 mm in cases without invasion, suggesting tumors exceeding 5 mm warrant heightened suspicion for deep extension (8, 30). This quantitative parameter provides objective criteria for escalating imaging evaluation and modifying treatment approaches. The real-time nature of UBM allows dynamic assessment with multiple scan orientations to determine maximum dimensions and extent (26, 29, 31).

This modality can identify areas of reduced echogenicity or thickening within scleral tissue that may indicate infiltration (29, 31, 32). Scleral invasion has profound therapeutic implications, often necessitating scleral grafting or plaque radiotherapy (6). However, clinical correlation remains essential, as smooth scleral indentation may occur with benign lesions while irregular thickening or destruction suggests malignant invasion. Integration of UBM findings with clinical features including tumor fixation, reduced mobility, and anterior chamber reaction enhances diagnostic accuracy.

### Intraocular extension

UBM excels in ciliary body visualization and anterior chamber assessment, providing detailed morphological evaluation impossible with conventional examination techniques and outperforming conventional B-scan ultrasonography (27, 29, 31). Signs of intraocular involvement include anterior chamber angle blunting, anterior segment mass effect, and ciliary body thickening (8, 27). The modality can detect small ciliary body lesions and characterize their extent, critical information for determining whether globe-salvaging therapy remains feasible or whether brachytherapy or enucleation is necessary.

Characteristic UBM features of intraocular OSSN extension include direct visualization of tumor tissue crossing the limbus into the anterior chamber, thickening of the ciliary body adjacent to areas of scleral involvement, and presence of hyperechoic deposits within the anterior chamber representing tumor cells or inflammatory debris (8).

The 4–5 mm penetration limitation restricts UBM’s ability to assess extensive orbital involvement, and structures beyond the ciliary body and anterior sclera cannot be adequately visualized (26–28). When UBM demonstrates extensive ciliary body involvement, marked scleral thickening, or significant anterior chamber mass effect, escalation to computed tomography (CT) or magnetic resonance imaging (MRI) is indicated to assess the full extent of posterior and orbital extension (26, 27, 33). Clinical features including proptosis, restricted motility, or palpable orbital masses mandate comprehensive orbital imaging regardless of UBM findings (28, 33).

### Complementary roles of UBM and AS-OCT

UBM and AS-OCT demonstrate synergistic rather than competitive capabilities in OSSN assessment, with each modality excelling in specific clinical scenarios (10, 34). In a direct comparative study of 200 anterior segment tumors, UBM demonstrated superior overall tumor visualization (69% versus 31%) and posterior margin resolution (74% versus 27%) compared to AS-OCT (34). This advantage proves particularly relevant for deeper lesions and assessment of posterior tumor extent. Conversely, AS-OCT excelled in anterior margin visualization and epithelial thickness quantification for OSSN diagnosis (12, 34).

The fundamental trade-off between the modalities involves resolution versus penetration depth (10, 14, 23). AS-OCT achieves superior axial resolution of 2–7 micrometers compared to UBM’s 25 micrometers, offering superior detail of superficial structures (11, 14). However, UBM visualizes deeper structures. It is preferred when there is concern for deep invasion: tumor height >5 mm (8, 30), thick nodular morphology including the nodulo-ulcerative variant (35), clinical signs such as reduced mobility/tissue fixation/anterior chamber reaction (8), recurrent OSSN, infeasible gonioscopy due to tumor size or location, or posterior shadowing on AS-OCT obscuring deep margins (14, 34).

Patient comfort strongly favors AS-OCT due to its non-contact nature, eliminating discomfort and potential complications from probe contact (11, 14). Image acquisition speed represents a significant AS-OCT advantage, enabling three-dimensional volumetric imaging and reducing motion artifacts (11). However, UBM’s real-time dynamic assessment remains valuable for angle evaluation and tumor positioning during examination (26, 31). **Table 1** summarizes the comparative features of each modality.

**Table 1:** Comparison of AS-OCT and UBM in OSSN.

Feature	AS-OCT	UBM
Contact	Non-contact	Contact (water-filled balloon)
Axial resolution	2-7 µm	~25 µm
Penetration depth	2–3 mm	4–5 mm
Epithelial detail	Excellent	Limited
Deep sclera	Limited	Good
Ciliary body	Not visualized	Excellent
Angle assessment	Good	Excellent
Patient comfort	High	Moderate
Main limitations	Posterior shadowing; cannot see behind iris	Lower resolution; contact; <5 mm depth
Indications	Superficial lesions; monitoring; surveillance	Deep extension; CB visualization; thicker lesions; shadowing on AS-OCT

**Figure 1:**
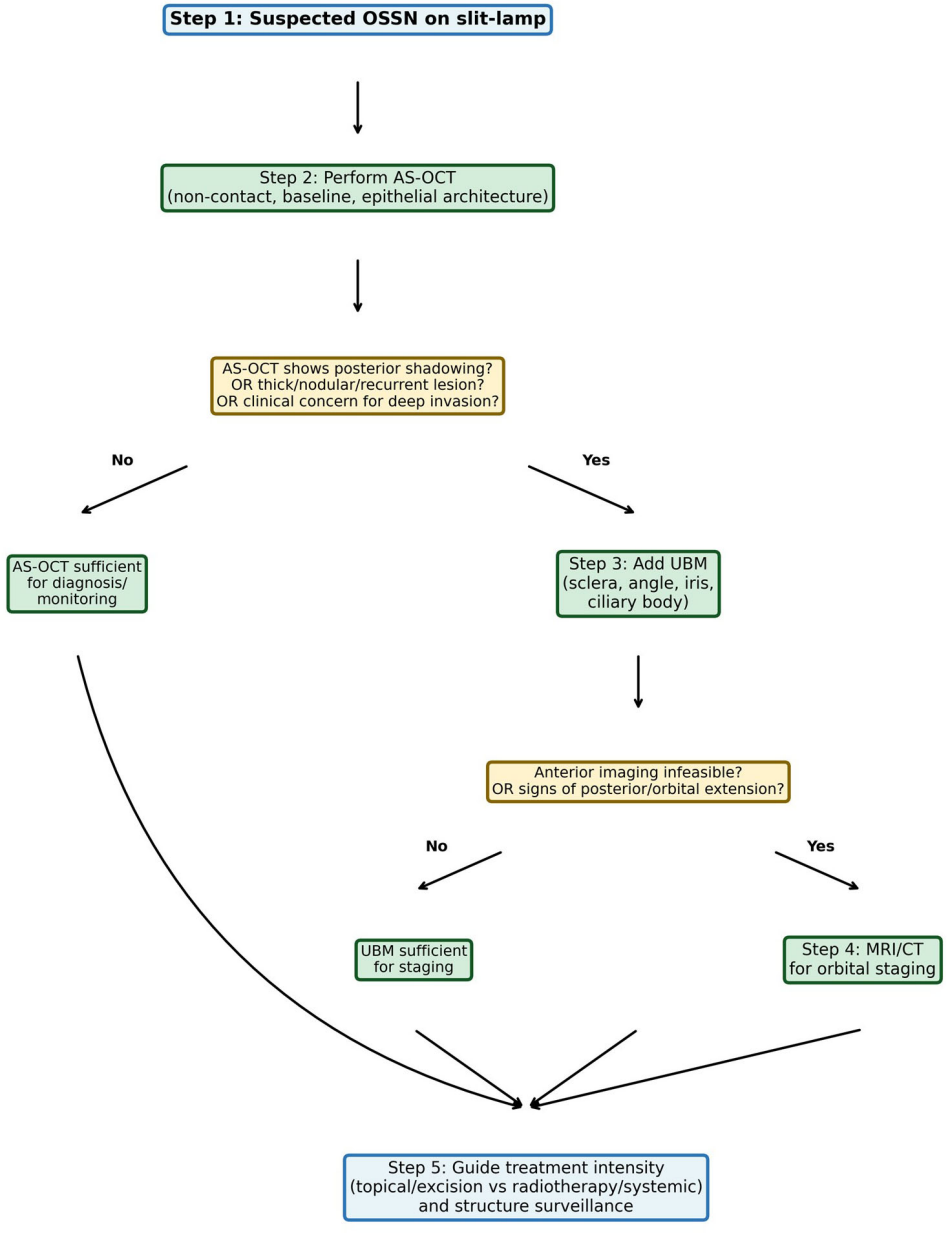
Stepwise imaging workflow for suspected OSSN.

AS-OCT as well as UBM may be infeasible in very large, irregular, painful, or highly elevated lesions. In such cases, and when clinical or imaging findings raise concern for posterior or orbital extension, cross-sectional imaging (MRI or CT) should be considered for staging. **Figure 1** presents a suggested stepwise imaging workflow for suspected OSSN.

## Illustrative cases

**Table 2** summarizes the clinical characteristics, imaging findings, and management of the presented cases.

**Table 2:** Summary of illustrative cases.

Case	Category	Imaging	Thickness	Key findings	Histopathology	Management
1	Recurrent SCC, intraocular	MRI, UBM	CB thickness- 1.43 mm (UBM)	CB thickening, angle/iris involved	Intraocular SCC	Excision + cryo + AMG+ Biopsy + brachytherapy + Cemiplimab
2	SCC, no deep extension	UBM	1.31 mm	Angle/iris/CB uninvolved	SCC, clear margins	Excision + cryo + AMG +MMC
3	High-grade CIN	AS-OCT	450-503 µm	Focal loss of cleavage plane	CIN 3, suspected minimal invasion	Excision + cryo + AMG + brachy
4	High-grade CIN	AS-OCT	370-420 µm	Clear cleavage plane	CIN 3, no invasion	Excision + cryo + AMG
5	CIN	AS-OCT, UBM	1.9 mm	AS-OCT shadowing; UBM: angle clear	CIN 2, no invasion	Excision + cryo + AMG
6	High-grade CIN	AS-OCT, UBM	0.58 mm	UBM: angle/CB uninvolved	CIN 3, no invasion	Excision + cryo + AMG
7	SCC	MRI	9 mm (MRI)	Extension toward medial rectus	Well-diff SCC	Complete excision+ cryo + AMG

AMG, amniotic membrane graft; CB, ciliary body; CIN, conjunctival intraepithelial neoplasia; cryo, cryotherapy; brachy, brachytherapy; MMC, Mitomycin C; SCC, squamous cell carcinoma; well-diff, well-differentiated.

### UBM for detection of deep invasion in SCC

#### Case 1

A 75-year-old man with type 2 diabetes mellitus (DM2), multiple basal cell carcinomas, and bilateral pseudophakia presented with persistent right-eye (OD) conjunctival redness for about two years that had worsened following cataract surgery. On slit-lamp, a wide temporal vascular conjunctiva-corneal lesion was noted. He underwent excisional biopsy with alcohol-assisted epithelial debridement and cryotherapy. Histopathology confirmed SCC with tumor-free surgical margins.

**Figure 2:**
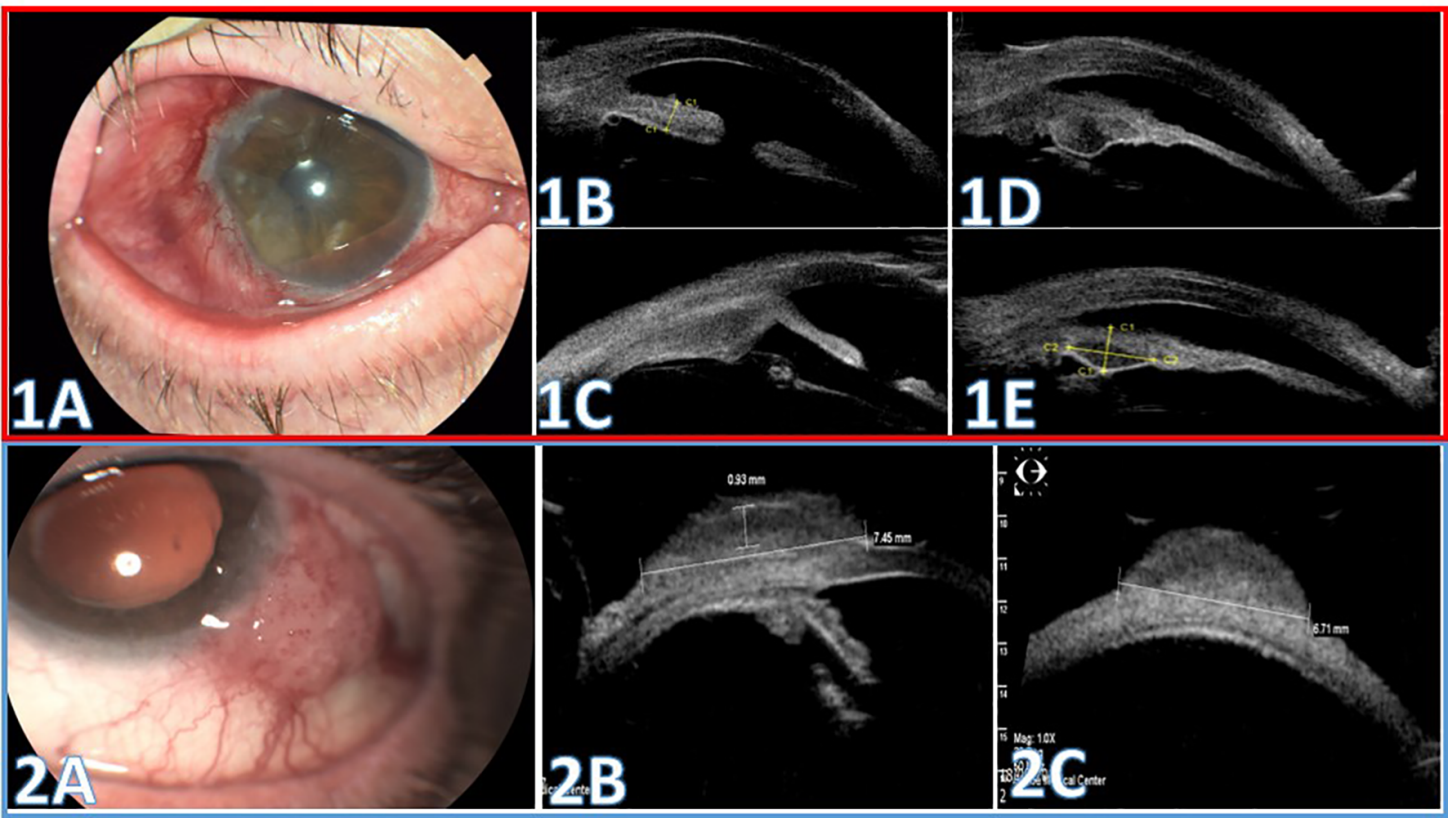
Ultrasound Biomicroscopy (UBM) scans for evaluation of anterior segment involvement in squamous cell carcinoma (SCC). Case 1(Red Box, **1A–1E**): **(1A)** Color photograph showing an extensive, vascular conjunctival-corneal lesion at 13 months from presentation. **(1B–D)** UBM of the anterior segment: 1B. Scan through the limbus and iris demonstrating iris thickening with angle involvement. **(1C)** Radial scan through the ciliary body demonstrating thickening. **(1D)** Horizontal scan along the iris root demonstrating iris base thickening with a cystic change. **(1E)** Same scan (as **1D**) at follow-up after brachytherapy demonstrating cyst contraction and reduced iris base thickening. Case 2 (Blue Box, 2A-2C): **(2A)** Color slit-lamp photograph shows a vascular gelatinous conjunctival-limbal lesion extending onto the corneal surface. **(2B, C)** UBM scans demonstrate a solid lesion overlying the limbus, anterior sclera and cornea. The maximal planimetric dimensions are 6.71 × 7.45 mm, and maximal thickness is 1.31 mm above the sclera. The anterior chamber angle, adjacent iris, and ciliary body are uninvolved.

At routine follow-up examinations, no clinical evidence of recurrence was noted until 10 months after the initial treatment, when clinical signs suggested recurrence. Topical 5-fluorouracil was recommended, but the patient was lost to follow-up and returned three months later with worsening of redness and irritation (**Figure 2, 1A**). MRI of the orbit excluded orbital invasion and did not delineate intraocular extension. UBM at this stage demonstrated anterior scleral wall thickening with extended ciliary body thickening, as well as angle and adjacent iris involvement (**Figures 2, 1B–D1**). Based on these findings demonstrating intraocular extension, the treatment plan was escalated to include brachytherapy and consider systemic immunotherapy. The patient underwent repeat biopsy this time with adjacent brachytherapy. Pathology confirmed intraocular invading SCC.

Given the clinical scenario, the patient was treated with adjuvant systemic Cemiplimab. On serial imaging, follow-up UBM showed reduced ciliary body thickness, a stable anterior scleral wall appearance, and no posterior extension (**Figure 2, 1E**). At 1-year follow-up, no recurrence was detected.

#### Case 2

An 81-year-old man with hypertension, ischemic heart disease (IHD) and bilateral pseudophakia, presented with a progressively enlarging red lesion in the left eye (OS) over several months. On slit-lamp, an extensive lesion extending to the corneal surface was noted (**Figure 2, 2A**). UBM showed a lesion involving the bulbar conjunctiva extending above the limbus onto the corneal surface. The maximal lesion dimensions measured 6.71 × 7.45 mm, with a maximal thickness of up to 1.31 mm above the scleral surface. The anterior chamber angle, adjacent iris, and ciliary body appeared normal, with no posterior extension (**Figures 2, 2B, C**). UBM exclusion of deep extension supported proceeding with local excision without escalation to brachytherapy. Histopathology confirmed SCC in the submitted specimen, with tumor-free margins and no scleral or intraocular invasion identified.

### AS-OCT for initial assessment in CIN

#### Case 3

**Figure 3:**
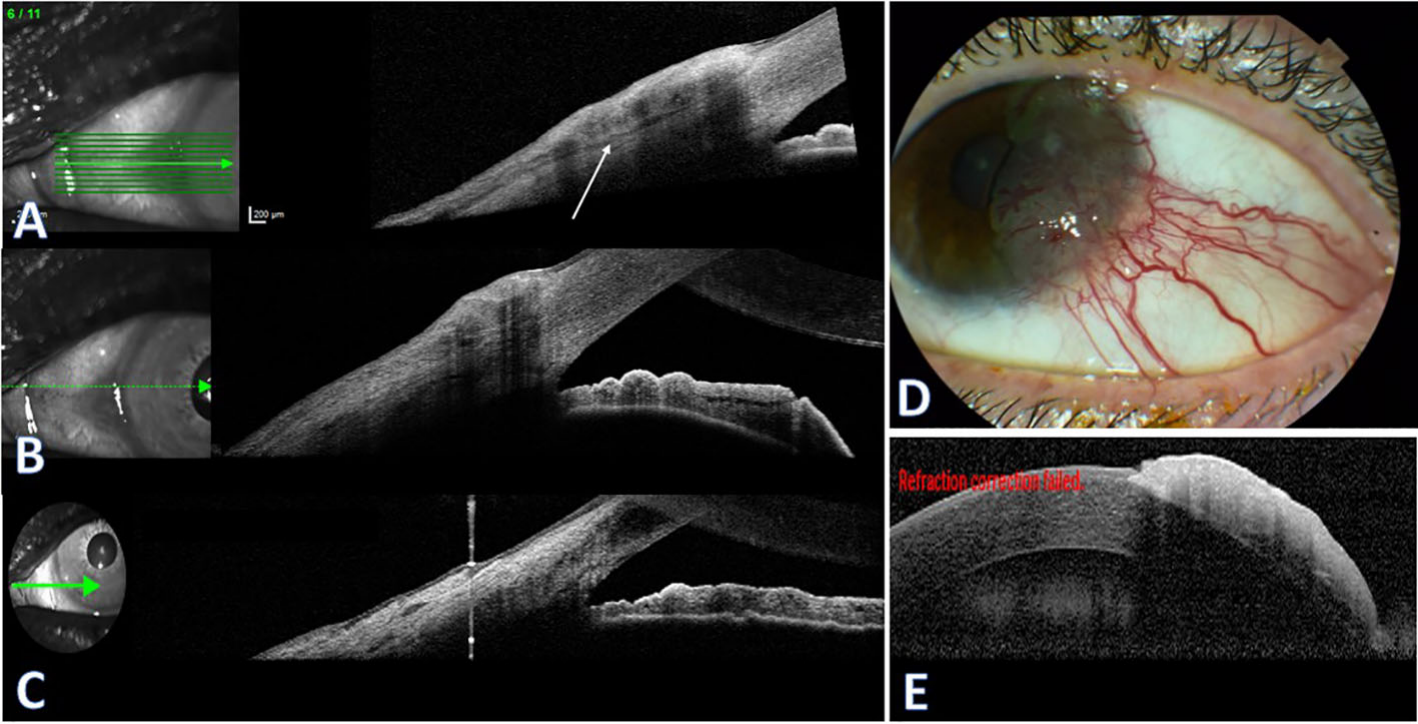
Anterior Segment Optical Coherence Tomography (AS-OCT) of high grade CIN. Case 3 **(A–C) (A)** AS-OCT shows a limbal-crossing lesion with epithelial thickening; a distinct separation plane from the underlying sclera and cornea is visible (white arrow) with posterior shadowing**. (B)** No separation plane is visible, with no involvement of the iris or anterior chamber angle. **(C)** Three years post-op: no recurrent lesion. Case 4 **(D, E). (D)** Color photograph showing a pseudo-pterygium fibrovascular lesion with prominent vascularization and feeder vessels. **(E)** AS-OCT demonstrating a similarly hyperreflective epithelial plaque with an abrupt transition zone and a clear separation plane from the underlying cornea.

A 60-year-old woman with a history of prior cerebral hemangioma resection presented with foreign body sensation OS. She had been evaluated elsewhere, where CIN was suspected, and was referred for further assessment. Clinical examination revealed a localized, slightly elevated limbal conjunctival-corneal lesion with mild vascularization, with no clinical evidence of intraocular extension. AS-OCT showed hyperreflective epithelial thickening (**Figure 3A**). Along the limbal margins, cross-sectional imaging demonstrated a distinct hyporeflective separation plane from the underlying scleral surface (**Figure 3A**), whereas in other scans this cleavage plane was lost (**Figure 3B**), raising concern for superficial stromal involvement. No anterior chamber or angle involvement was evident on clinical examination or with AS-OCT.

The lesion was completely excised with alcohol-assisted epithelial debridement and double freeze cryotherapy, with immediate ocular surface reconstruction using an amniotic membrane graft. Histopathology confirmed high-grade conjunctival intraepithelial neoplasia (CIN 3) with foci suspicious for minimal stromal invasion. In view of the high-grade pathology and concern for residual microscopic disease, brachytherapy was subsequently performed. At 3 years of follow up, no recurrence was seen clinically or on AS-OCT (**Figure 3C**).

#### Case 4

A 65-year-old man with hypertension and DM2 was referred for a suspicious conjunctival-corneal lesion OS. He reported remote ocular trauma to the same eye 20 years earlier. Clinical examination revealed a pseudo-pterygium-like fibrovascular plaque with engorged, tortuous feeder vessels and subtle corneal extension, without clinical evidence of intraocular involvement (**Figure 3D**). AS-OCT demonstrated a thickened epithelial lesion draping onto the corneal surface with an abrupt transition zone and a clearly demarcated corneal interface (**Figure 3E**). No anterior chamber or angle involvement was identified. The lesion was completely excised with double freeze cryotherapy and ocular surface reconstruction using an amniotic membrane graft. Histopathology showed high-grade conjunctival intraepithelial neoplasia (CIN 3) without stromal invasion. The patient has been followed for 6 years with no recurrence.

### Complementary UBM imaging when AS-OCT is limited

#### Case 5

**Figure 4:**
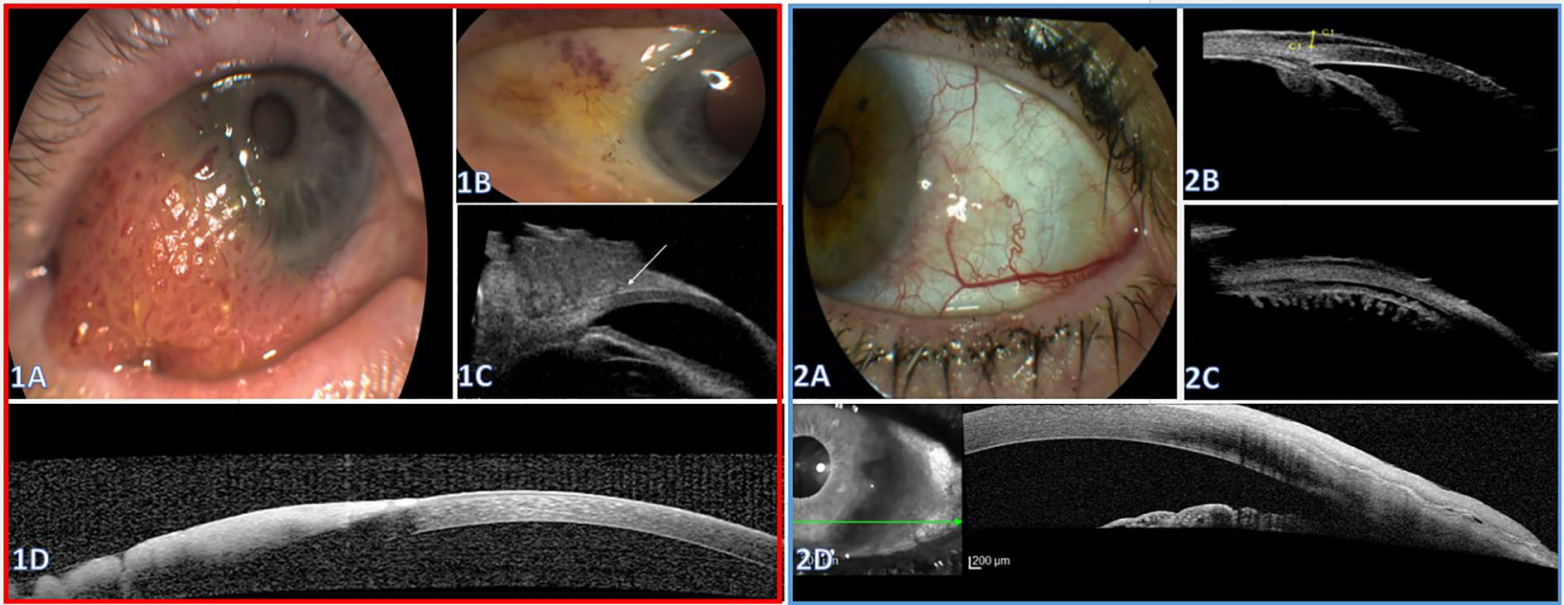
Suspected OSSN with complementary AS-OCT and UBM. Case 5 (Red Box, **1A–D**) **(1A)** Color photograph showing an elevated thick gelatinous conjunctival-corneal mass. **(1B)** One-month post-excision color photograph demonstrating a quiet ocular surface without residual lesion. **(1C)** Ultrasound Biomicroscopy (UBM) showing a lesion elevated over the limbus without corneal invasion; a clear separation plane from the anterior cornea is visible (arrow). The angle and adjacent iris appear uninvolved. **(1D)** Anterior Segment Optical Coherence Tomography (AS-OCT) demonstrating a hyperreflective, thickened epithelial lesion extending onto the cornea with marked posterior shadowing that precludes visualization of the underlying corneal layers. Case 6 (Blue Box, **2A–D**): **(2A)** Color photograph shows a pseudo-pterygium-like fibrovascular lesion with a prominent feeder vessel at the conjunctival-limbal junction and subtle corneal extension. **(2B, C)** UBM, radial scan (B) and transverse scan (C) through the lesions, shows a hypoechoic lesion of 0.58-mm-thickness overlying the anterior sclera and limbus with an uninvolved angle, iris, and ciliary body (B) and uninvolved ciliary processes (C). **(2D)** AS-OCT shows a hyperreflective epithelial plaque draping across the limbus and anterior cornea. The posterior interface is largely discernible with hyperreflective line.

A 90-year-old man with a history of multiple excised cutaneous lesions, including SCC, was referred for evaluation of a conjunctival-corneal mass suspicious for OSSN. Slit-lamp examination showed a red gelatinous lesion with vascular dilatations OS (**Figure 4, 1A**). AS-OCT demonstrated a hyperreflective epithelial mass with marked surface reflectivity causing complete posterior shadowing, which precluded assessment of the deeper interface and posterior margin (**Figure 4, 1D**). UBM delineated a bulky lesion draping over the sclera and extending onto the cornea. The anterior chamber angle, ciliary body and sclera did not appear involved, although evaluation was limited by lesion thickness and acoustic attenuation. A thin cleavage plane was appreciable between the anterior corneal surface and the superficial portion of the mass (**Figures 4, 1B, C**). The imaging results supported proceeding with excision alone.

The lesion was excised completely with cryotherapy and amniotic membrane transplantation. Histopathology of the primary specimen demonstrated CIN grade 2 (moderate epithelial dysplasia) without stromal invasion.

#### Case 6

A 78-year-old woman was referred for a pseudo-pterygium-like conjunctival-limbal lesion with a prominent feeder vessel (**Figure 4, 2A**). AS-OCT demonstrated a hyperreflective epithelial plaque over the corneal, limbal, and anterior scleral surfaces. A mostly well-defined posterior interface was appreciable, although shadowing produced locally indistinct borders at the lesion margins (**Figure 4, 2D**). Due to signal shadowing, the angle was suboptimally visualized on AS-OCT, UBM was performed showing a solid lesion over the limbus with corneal draping. Maximal thickness measured up to 0.58 mm above the scleral surface. The anterior chamber angle, adjacent iris, and ciliary body and processes appeared normal, with no posterior extension (**Figures 4, 2B, C**), supporting local excision. Histopathology showed high-grade conjunctival intraepithelial neoplasia (CIN 3) without stromal invasion; the stroma demonstrated subepithelial elastotic degeneration consistent with pterygium.

### MRI for extensive disease when anterior imaging is infeasible

#### Case 7

**Figure 5:**
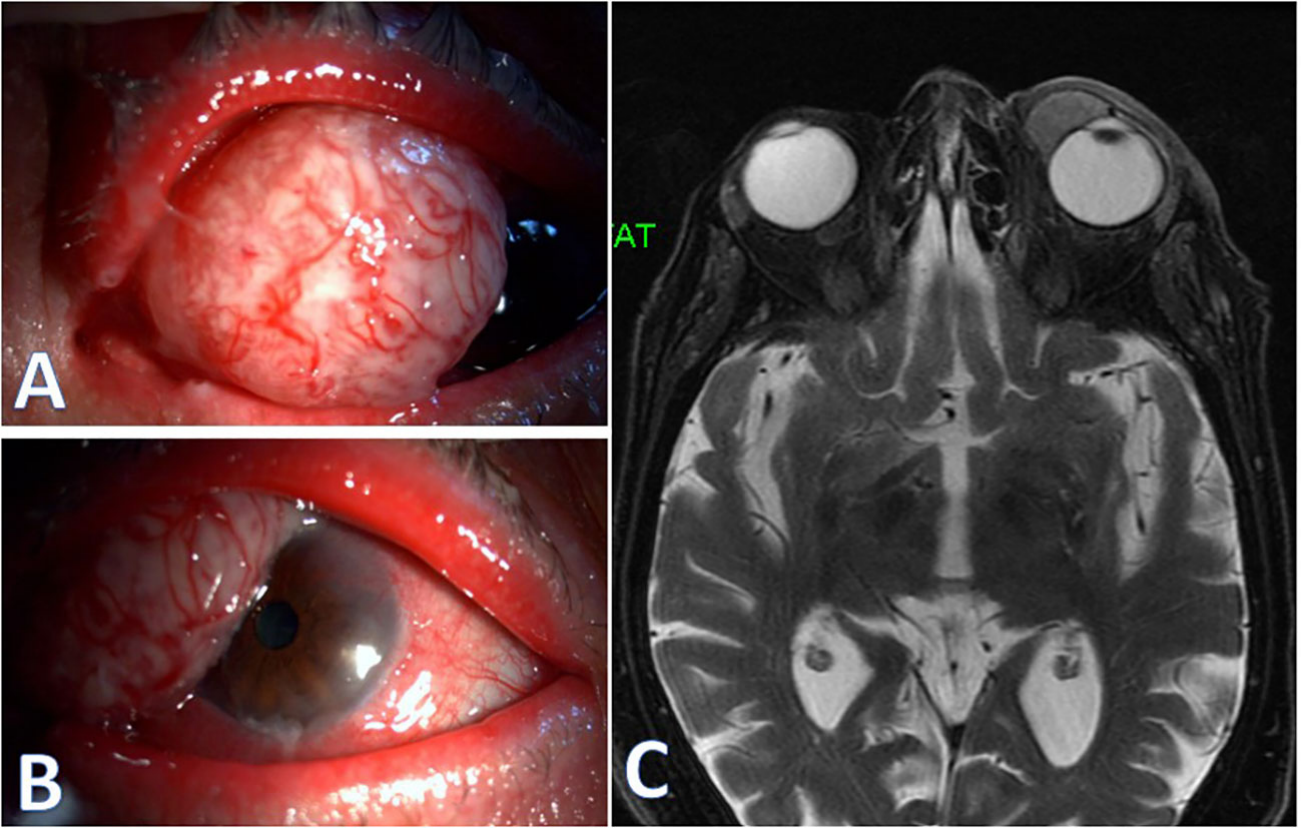
**(A, B)** Color slit-lamp photographs showing a large vascular mass covering approximately half of the corneal surface. **(C)** Orbital magnetic resonance imaging (MRI) with an arrow marking the lesion along the anterior medial globe wall, showing posterior extension toward the medial rectus region.

An 86-year-old woman with DM2 and hypertension, presented with a large nasal conjunctival-corneal mass obscuring approximately half of the corneal surface. Owing to the lesion’s height and configuration, AS-OCT and UBM were not feasible as well as angle examination (**Figures 5A, B**). Therefore, orbital MRI was selected to evaluate possible deep and orbital extension. Preoperative MRI demonstrated a large lesion along the anterior globe wall with posterior extension toward the medial rectus region, without intraorbital invasion (**Figure 5C**). The mass was then completely excised, and histopathology confirmed well-differentiated SCC.

### Strengths and limitations

This review offers several practical contributions: (1) a stepwise imaging workflow integrating AS-OCT, UBM, and cross-sectional imaging based on clinical indications (**Figure 1**); (2) a comparative summary of the advantages and limitations of each modality (**Table 1**); and (3) real-world cases illustrating the clinical utility of each imaging modality.

Several limitations should be acknowledged. This is a convenience sample from a single tertiary center and does not represent a consecutive cohort. Imaging selection was clinician-driven, which may introduce selection bias. Measurements were performed on different imaging modalities depending on clinical indication, limiting direct comparisons. Although published literature suggests tumor height thresholds (e.g., >5 mm) for predicting deep invasion, quantitative measurements in this series were not systematically applied to treatment decisions; rather, imaging served primarily to confirm or exclude deep extension qualitatively. Therefore, this review is intended to illustrate clinical applications rather than to assess diagnostic accuracy or establish imaging thresholds.

## Summary

OSSN can involve multiple anterior segment tissues, and clinical examination alone often struggles to define depth of invasion and involvement of adjacent structures. Imaging adds a decisive layer for staging and management. AS-OCT offers non-contact, high-resolution assessment of epithelial architecture that supports initial diagnosis, treatment monitoring, and early recurrence recognition. UBM provides approximately 4–5 mm penetration and visualizes the anterior sclera, angle, and ciliary body, helping to uncover deep or clinically occult extension. By integrating these complementary strengths into a stepwise workflow, clinicians can improve staging accuracy and align treatment intensity with true disease extent, while reserving CT or MRI for suspected orbital involvement.

## Data Availability

The original contributions presented in the study are included in the article. Further inquiries can be directed to the corresponding author.
